# Whole-genome sequencing of *Chromobacterium subtsugae* strains exhibiting toxicity to *Drosophila melanogaster*

**DOI:** 10.1128/mra.00127-24

**Published:** 2024-04-30

**Authors:** Madison M. Clark, Nichole A. Broderick

**Affiliations:** 1 Department of Biology, Johns Hopkins University, Baltimore, Maryland, USA; University of Maryland School of Medicine, Baltimore, Maryland, USA

**Keywords:** *Drosophila*, host-pathogen, infection model, gut homeostasis

## Abstract

*Chromobacterium subtsugae* exhibits toxicity to *Drosophila melanogaster*, providing a new infection model to study host homeostasis. Previous studies using pathogen models have proven to be a useful tool to understand host physiology. Here, we report on the whole-genome sequences of these microbes obtained from short and long reads.

## ANNOUNCEMENT

*Chromobacterium subtsugae* is known for its ability to produce the purple-pigmented metabolite violacein and exhibits toxicity to *Drosophila melanogaster* (1). *C. subtsugae* ATCC 31532 was isolated from a soil sample grown on nutrient agar at 25°C for 72 hours and was initially classified as *Chromobacterium violacein* SC 11,378 (2). However, this strain has been reclassified as *Chromobacterium subtsugae* (3). *C. subtsugae ∆vioS* (*∆vioS*), formerly CV017, was derived from ATCC 31532 and contains a transposon insertion resulting in the overproduction of violacein (4).

Both strains were grown on Luria Broth (Invitrogen) agar plates for 48 hours at 30°C before they were sent to SeqCoast (Portsmouth, NH) for DNA extraction and sequencing. DNA was extracted using the MagMAX Microbiome Ultra Nucleic Acid Isolation Kit (Applied Biosystems) and sequenced with Illumina and Oxford Nanopore Technologies. Illumina sequencing samples were prepared with the Illumina DNA Prep Tagmentation Kit. Amplicons < 500 bp were depleted without DNA fragmentation. Sequencing was performed on the Illumina NextSeq2000 platform using a 300-cycle flow cell kit to produce 2 × 150 bp paired reads. One to two percent of PhiX control was spiked into the run to support optimal base calling. Read demultiplexing, read trimming, and run analytics were performed using DRAGEN v3.10.12 (5). Default parameters were used for all software unless otherwise specified. This resulted in 13,205,486 reads (36–151 bp) for ATCC 31532 and 21,435,796 reads (35–151 bp) for *∆vioS*. Quality control was performed using FastQC v1.2.2 (6). Illumina adapters were removed using Trimmomatic v1.2.14 (7).

Oxford Nanopore sequencing samples were prepared using the Oxford Nanopore SQK-NBD114 native barcoding kit. Fragments < 25 kb were depleted without DNA fragmentation. Long Fragment Buffer was used to promote longer read lengths. Sequencing was performed on the GriDION platform using a FLOW-MIN114 Spot-ON Flow Cell, R10 version with a translocation speed of 400 bps. Base calling was performed on the GridION v23.07.5 with Guppy v7.0.9 (8). Nanopore adapters were removed using PoreChop v.0.2.4 (9), resulting in 215,056 reads (*N*_50_ = 4,875 reads) for ATCC 31532 and 646,016 reads (*N*_50_ = 3,888 reads) for *∆vioS*.

Hybrid genomes using Illumina and Nanopore sequences were generated using KBase (10). Assembly was performed using Unicycler v0.4.8 (11) through the following steps: (i) contigs were generated using SPAdes v3.15.3 (12, 13), (ii) reads were mapped with Bowtie2 v2.4.2 (14) and SAMtools v1.11 (15), and (iii) assemblies were polished with Pilon v1.24 four times (16). The ATCC 31532 assembly is 4,760,091 bp long (105× coverage), has a GC content of 64.7%, and consists of 1 contig (*N*_50_ = 4,760,091 bp). The *∆vioS* assembly is 4,765,196 bp long (105× coverage), has a GC content of 64.7%, and consists of 1 contig (*N*_50_ = 4,765,196 bp). Each assembly was determined to be circular and was rotated to start at DnaA on the forward strand using Unicycler v0.4.8 (11). *∆vioS* is ~5 kb larger than ATCC 31532 due to the transposon insertion. Genomes were annotated using PGAP v6.5 (17).

These high-quality genomes will aid in understanding host-microbe interactions to identify innovative mechanisms to limit microbial damage during infection.

## Data Availability

*Chromobacterium subtsugae* ATCC 31532 data are under GenBank accession CP142381, and raw sequencing reads are under SRA accession numbers SRR27256803 (Illumina) and SRR27256802 (Nanopore). Chromobacterium subtsugae ∆vioS data are under GenBank accession CP143257, and raw sequencing reads are under SRA accession numbers SRR27256753 (Illumina) and SRR27256752 (Nanopore).
